# Landscape patterns of species-level association between ground-beetles and overstory trees in boreal forests of western Canada (Coleoptera, Carabidae)

**DOI:** 10.3897/zookeys.147.2098

**Published:** 2011-11-16

**Authors:** J. A. Colin Bergeron, John R. Spence, W. Jan A. Volney

**Affiliations:** 1 Department of Renewable Resources, University of Alberta, 751 General Services Building, Edmonton, Alberta, Canada T6G 2H1; 2 Natural Resources Canada, Northern Forestry Centre, 5320 122 Street Northwest, Edmonton, Alberta, Canada T6H 3S5

**Keywords:** Landscape ecology, Carabidae, Trees, Forest canopy mosaic, Biodiversity surrogates, Regional conservation

## Abstract

Spatial associations between species of trees and ground-beetles (Coleoptera: Carabidae) involve many indirect ecological processes, likely reflecting the function of numerous forest ecosystem components. Describing and quantifying these associations at the landscape scale is basic to the development of a surrogate-based framework for biodiversity monitoring and conservation. In this study, we used a systematic sampling grid covering 84 km^2^ of boreal mixedwood forest to characterize the ground-beetle assemblage associated with each tree species occurring on this landscape. Projecting the distribution of relative basal area of each tree species on the beetle ordination diagram suggests that the carabid community is structured by the same environmental factors that affects the distribution of trees, or perhaps even by trees *per se*. Interestingly beetle species are associated with tree species of the same rank order of abundance on this landscape, suggesting that conservation of less abundant trees will concomitantly foster conservation of less abundant beetle species. Landscape patterns of association described here are based on characteristics that can be directly linked to provincial forest inventories, providing a basis that is already available for use of tree species as biodiversity surrogates in boreal forest land management.

## Introduction

Although some ground-beetle species are recognized as forest habitat specialists (Lindroth 1961-69, Niemelä et al. 1992), the potential influence of ecological linkage between carabids and particular tree species has not been much studied. Adult or larval carabids may use deadwood as shelter, oviposition or overwintering sites (Goulet 1974). They may feed on prey or vegetative items that are associated with trees and the related forest floor (e.g., Koivula et al. 1992), or use particular understory forest plants as food or shelter. Some carabid species may require a certain amount of shade or specific forest floor conditions that are best provided by certain tree species. Ground-beetle assemblages are known to vary generally with stand canopy cover (Niemelä et al. 1992; Pearce et al. 2003; Gandhi et al. 2008; Work et al. 2010), and it is frequently assumed that this reflects strong relationships between tree cover and local edaphic conditions (Perry et al. 2008). Nonetheless, connections of these patterns of association to the indirect ecological interactions mentioned above remain largely undescribed. We do not know much about the specific elements of forest stands that affect the structure of carabid assemblages.

Associations between forest composition and ground-beetle species could involve a host of indirect processes, reflecting the ecological interactions of numerous forest ecosystem components (e.g., Allegro and Sciaky 2003). In a practical sense, modelling patterns of association between specific tree species and ground-beetles would reflect a more restricted subset of these processes but still include the state of many biotic and abiotic elements that are difficult or impossible to observe directly. Beetles of the family Carabidae are a group of choice for forest health assessment at local scales (Work et al. 2008), given the well developed taxonomic resources to facilitate species-level identification, the simplicity of sampling them, the fact that suitable habitat (i.e., the litter and upper soil layers) is not removed by forest harvest, and the microscale at which carabids interact with their environment making them sensitive indicators of change. Understanding their associations with particular trees species could increase their usefulness as indicators of human impact on forest landscapes. More importantly, as is our focus here, if such relationships between carabid and tree species can be defined, commonly available data about forest inventories could provide surrogates for at least this element of biodiversity. This, in turn, would be quite valuable in the context of monitoring requirements associated with forest certification and sustainable forest management.

As it is impossible to appropriately assess biodiversity of entire forested landscapes, implementation of practical conservation strategies must be based to some extent on biodiversity surrogates (Spence et al. 2008). Trees are easier to survey over large areas than are most of the small and often cryptic organisms that constitute the majority of biodiversity. Thus, trees have the potential to be excellent biodiversity surrogates for forest land, if their spatial arrangement shows concomitant variation with that of other living organisms. In fact, tight associations between tree and beetle species are central to the first well known scientifically based estimate of the global number of insect species (Erwin 1982) and community structure of herbivorous arthropods is well known to differ among tree species (Southwood et al. 1982). Knowing relationships between tree species cover and the ground-beetle community living in a forest may support useful broad-scale characterization of biodiversity and ecosystem function based on simple elaboration of tree species distribution. Mapping tree species distributions is easily achieved via remote sensing. Knowledge of relationships between these distributions and biodiversity could prompt more effective and efficient conservation efforts over wide areas by ensuring maintenance of suitable volumes of non-commercial and rare tree species on managed landscapes.

In the boreal forest, studies of carabid-tree relationships have been mainly based on stand-level categorization of canopy cover (e.g., conifer vs deciduous or spruce dominated vs aspen dominated) (e.g., Niemelä et al. 1992; Pearce et al. 2003; Jacobs et al. 2008; Work et al. 2010) and thus do not consider the individual contribution of each tree species present. We hypothesize that many insects, including ground-beetles, perceive the forested landscape as a combination of multiple spatial gradients that supply the resources they require. As such, we predict that the structure of carabid assemblages should change along a forest transect in relation to the relative importance of every tree species included in providing resources or fostering conditions used by the beetles. In this context, ground-beetle assemblages might not be best viewed as Clementsian entities tightly associated to certain environmental conditions, but rather as one big Gleasonian community in which species abundances vary independently with environmental conditions. In this study, we assess patterns of association between ground-beetles and mature tree species on a northern forested landscape level using a systematic sampling design.

## Materials and methods

### Study area

The study was conducted at the EMEND (Ecosystem Management Emulating Natural Disturbance) research site in the boreal mixedwood forest of northwestern Alberta, Canada. The approximate project centre is at 56°46'13"N, 118°22'28"W, ~90 km northwest of Peace River, (see Work et al. 2004 for the location of the site on Alberta map). The elevation varies between 677 and 880 m asl. The forest is a varying mixture of *Picea glauca* (Moench), *Populus tremuloides* Michx., *Populus balsamifera* L., combined with *Picea mariana* (Mill.) BSP and *Larix laricina* (Du Roi) K. Kock in wetter sites, with occasional *Abies balsamea* (L.) Mill., *Betula papyrifera* Marsh and *Pinus contorta* Loudon, representing the boreal-montane transitional nature of the lower foothill ecoregion. *Viburnum edule* (Michx.) Raf., *Rosa acicularis* Lindl., *Sherpherdia canadensis* (L.) Nutt., *Alnus crispa* (Ait.) Purch, *Alnus tenufolia* Nutt., and *Ledum groenlandicum* Oeder are common forest understory plants, and open meadows, fens, and bogs sometimes dominated by willow (*Salix* spp.) or alder (*Alnus* spp.) shrubs are interspersed on this typical boreal landscape.

### Sampling design

In the summer of 2002, a systematic grid of 200 sites covering 84 km^2^ (Fig. 1) of forested land was established in order to describe landscape patterns of ecological association between ground-beetles and overstory trees. At every site, we recorded diameter at breast height (dbh) and species identity for the 25 stems over 5cm dbh closest to our sample centre. We also documented the dbh and species of one stem for tree species present within a 50 meters radius but not recorded among the 25 stems encompassed in the original plots. This allowed us to consider the potential influence of proximal tree species on the beetle assemblages that were not included among the 25 stems. Drainage was recorded from two soil pits dug at 10 meters east and west from the plot centre. Drainage was categorized using a system of 11 classes, as modified from Beckingham et al. (1996), to classify sites that showed characteristics of two adjacent classes. In the systematic grid, sites were located approximately 640 m apart. However, grid position of sites was constrained by two conditions: all sites had to be at least 40 meters from any 1) anthropogenic disturbance or 2) natural area without trees larger than 5 cm dbh. These conditions focused our study on possible relationships between the epigaeic fauna and mature forest heterogeneity. As is typical of boreal landscapes, the EMEND site includes wet areas, peatlands and much local variation in forest cover. Therefore, in 57 cases where the above criteria were not met, the actual site location was moved further than 40 meters and placed in the nearest forest stand. Thus, the sampling grid illustrated in Fig. 1 is not perfectly regular. Two sites in an extensive harvest block were omitted because relocated sites would have been further from the original gridpoint than the nearest neighboring site.

The following summer (2003), we installed three pitfall traps in each of the same 200 sites (600 traps total). Traps were located at 0º (North), 120°, and 240° on a circle 15 m in diameter centred on the previously established site. Pitfall traps were a plastic cup (11 cm diameter by 13 cm depth), containing a plastic inner collecting cup and covered by a 14 cm^2^ of plywood supported over the trap by two nails (see Spence and Niemelä 1994). Traps were operated during the frost-free season (i.e., from early May until the end of August), providing a potential total of 100 trapping days. We also sampled the epigaeic fauna in the two grid sites omitted from tree samples that were located in large harvest blocks and established an extra trapping site in the only naturally burnt forest encountered on the landscape. Four sites established in year 1 were not sampled for beetles because they were harvested over the intervening winter. Hence, we collected beetle data in a total of 197 sites (Fig. 1). All beetles from the family Carabidae were identified to the species level using Lindroth (1960-1969). Nomenclature follows Bousquet (1991), and voucher specimens are deposited in the E. H. Strickland Entomological Museum (University of Alberta) in Edmonton, Canada and the Spence Laboratory Collection.

**Figure:**
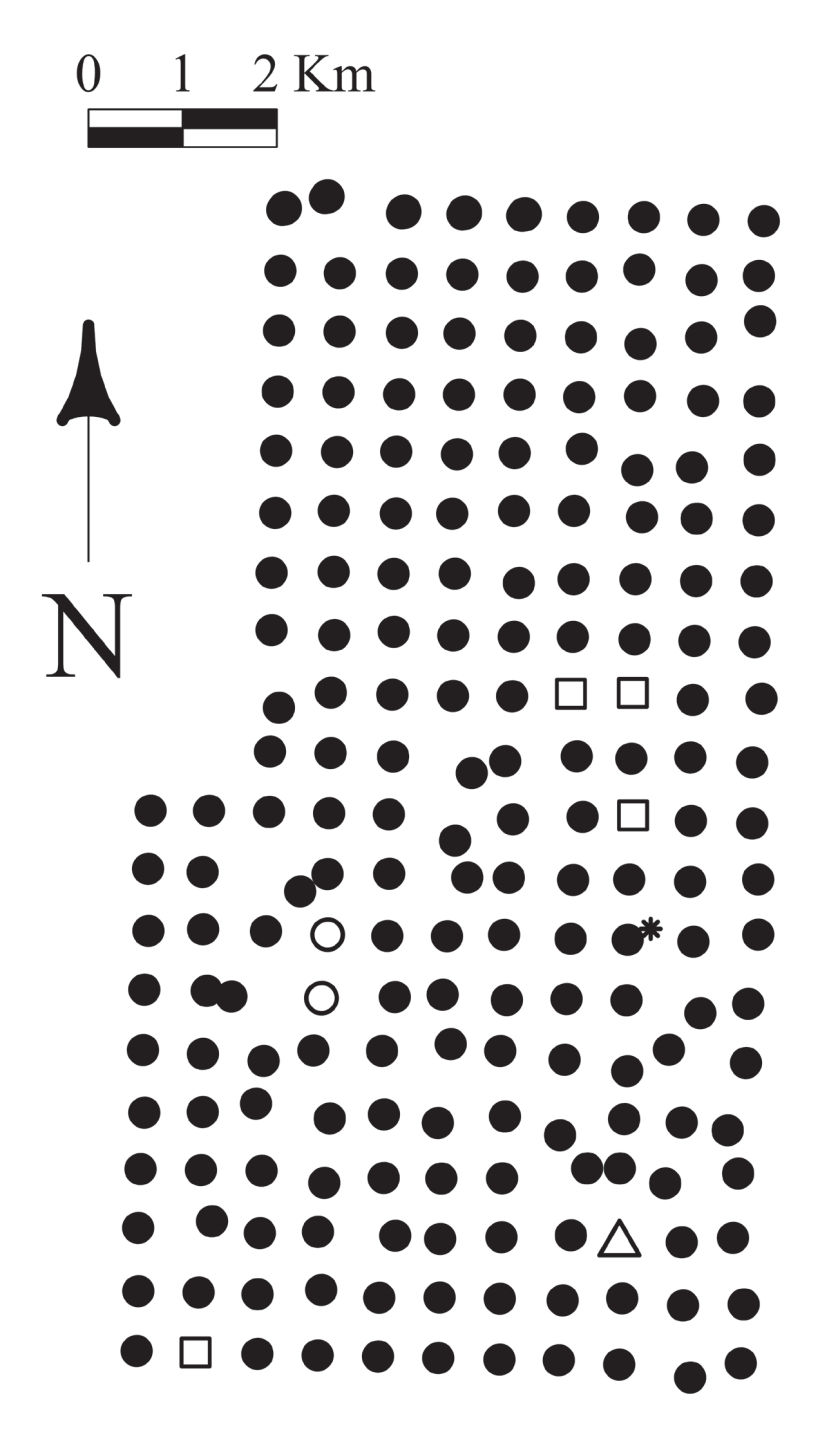
**Figure 1.** Map of the sampling sites. Squares represent destroyed sites, open circles represent harvested sites, triangle represents the outlier, and the star represents the burnt site.

### Statistical analyses

As a first step in linking within-stand canopy heterogeneity to ground-beetle assemblages, we calculated the relative basal area for each tree species in every site. Basal area is directly related to canopy cover (Spurr 1960) and, thus, appropriately represents the canopy influence of each tree species at a particular site. For each site, beetles from the three traps were pooled and their abundance divided by the sum of effective trapping days. This procedure allowed us to standardize beetle catches in relation to effective sampling effort. Although we report the abundance of beetle species collected in all 197 sites for entomological interest, the following statistical treatments were based only on the 194 grid sites located in forest with mature trees. This allowed us to focus on the effect of canopy heterogeneity on the beetle community.

In order to visualize how the assemblage of ground-beetles was arrayed on the EMEND landscape, we performed a non metric multidimentional scaling (NMS) ordination of 194 sites based on the standardized abundance of the beetle species. We used the Bray-Curtis dissimilarity index to build the distance matrix and chose the highest number of dimensions providing a reduction of five in the stress (McCune and Grace 2002). The NMS was calculated using a random start configuration, with a maximum of 20 iterations on the real data. Initial inspection of the resulting ordination showed that one site was a clear outlier and the ordination was recalculated without this site. This site was located in a small ellipse of trees left in a harvest block and had been disturbed in a similar way as the 3 other sites that had been previously removed from the analysis. For the 16 most abundant beetle species and all the tree species, we calculated centroids to represent average location of the species' position in the ordination space. Contribution of each site to centroid calculation was weighted by the standardized abundance (or relative abundance in case of trees) of this species in each site. The general relation between drainage and ground-beetle assemblage was illustrated by projecting vectors of influence on the ordination diagram.

Beetle assemblage response to forest canopy was more clearly illustrated by plotting the relative basal area of tree species for every site on the beetle ordination diagram. This procedure was undertaken because interesting ecological trends were obscured by the sole use of centroids or vectors. Centroid calculation, vector projection, and ordination were performed using the vegan package (Oksanen et al. 2011) within the R statistical language (R development core team 2010).

## Results

We collected and identified 9,845 individual ground-beetles representing 48 species (Table 1). *Stereocerus haematopus* (Dejean), *Calathus advena* (LeConte) and *Pterostichus adstrictus* Eschscholtz accounted for over 70% of catches, and together with the next seven most abundant species [*Platynus decentis* (Say), *Calathus ingratus* Dejean, *Pterostichus punctatissimus* (Randall), *Agonum retractum* LeConte, *Trechus chalybeus* Dejean, *Patrobus foveocollis* (Eschscholtz), and *Pterostichus brevicornis* (Kirby)], accounted for 95% of the beetle catch. Nine beetle species (*Agonum cupreum* Dejean, *Amara laevipennis* Kirby, *Amara patruelis* Dejean, *Cymindis unicolor* Kirby, *Dyschirius hiemalis* (Bousquet), *Elaphrus clairvillei* Kirby, *Notiophilus semistriatus* Say, *Poecilus lucublandus* (Say) and *Trichocellus mannerheimii* (R.F. Sahlberg)) were trapped only in the 4 disturbed sites.

**Table 1.** Species of the family Carabidae collected in 197 sites during the summer of 2003 in boreal Alberta, Canada. n= sample size.

Species	Catches	% catch	n sites
*Stereocerus haematopus* (Dejean)	2830	28.63	164
*Calathus advena* (LeConte)	2665	26.96	150
*Pterostichus adstrictus* Eschscholtz	1427	14.44	127
*Calathus ingratus* Dejean	607	6.14	118
*Platynus decentis* (Say)	529	5.35	114
*Pterostichus punctatissimus* (Randall)	421	4.26	100
*Agonum retractum* LeConte	349	3.53	78
*Trechus chalybeus* Dejean	285	2.88	78
*Patrobus foveocollis* (Eschscholtz)	223	2.26	76
*Pterostichus brevicornis* (Kirby)	172	1.74	71
*Carabus chamissonis* Fisher von Waldheim	159	1.61	69
*Agonum gratiosum* (Mannerheim)	48	0.49	20
*Platynus mannerheimii* (Dejean)	34	0.34	18
*Pterostichus pensylvanicus* LeConte	23	0.23	17
*Trechus apicalis* Motschulsky	14	0.14	12
*Agonum sordens* Kirby	11	0.11	6
*Nebria gyllenhali* Kirby	8	0.08	6
*Bembidion grapii* Gyllenhal	7	0.07	5
*Agonum cupreum* Dejean	7	0.07	4
*Notiophilus directus* Casey	6	0.06	4
*Synuchus impunctatus* (Say)	6	0.06	4
*Trichocellus mannerheimii* (R.F. Sahlberg)	5	0.05	4
*Pterostichus riparius* (Dejean)	5	0.05	3
*Trichocellus cognatus* (Gyllenhal)	5	0.05	3
*Amara erratica* (Duftschmid)	5	0.05	2
*Patrobus septentrionis* Dejean	4	0.04	2
*Calosoma frigidum* Kirby	3	0.03	2
*Elaphrus clairvillei* Kirby	2	0.02	2
*Notiophilus borealis* T.W. Harris	2	0.02	2
*Cymindis unicolor* Kirby	2	0.02	2
*Loricera pilicornis* (Fabricius)	2	0.02	1
*Bembidion rupicola* (Kirby)	2	0.02	1
*Amara lunicollis* Shiødte	2	0.02	1
*Amara laevipennis* Kirby	1	0.01	1
*Agonum placidum* (Say)	1	0.01	1
*Agonum superioris* Lindroth	1	0.01	1
*Amara littoralis* Mannerheim	1	0.01	1
*Amara patruelis* Dejean	1	0.01	1
*Badister obtusus* LeConte	1	0.01	1
*Dyschirius hiemalis* (Bousquet)	1	0.01	1
*Elaphrus lapponicus* Gyllenhal	1	0.01	1
*Harpalus fulvilabris* Mannerheim	1	0.01	1
*Harpalus laevipes* Zetterstedt	1	0.01	1
*Miscodera arctica* (Paykull)	1	0.01	1
*Notiophilus semistriatus* Say	1	0.01	1
*Poecilus lucublandus* (Say)	1	0.01	1
*Sericoda quadripunctata* (DeGeer)	1	0.01	1
*Elaphrus americanus* Dejean	1	0.01	1
			
Total	9885		
n species	48		

Among tree species, *Picea glauca* was found in the highest number of sites followed by *Populus tremuloides*, *Picea mariana*, *Populus balsamifera*, *Abies balsamea*, and *Larix laricina* (Table 2). *Picea glauca* was also the most abundant species, and *Picea mariana* was the second most abundant, accounting for twice as many stems as *Populus tremuloides*, even if *Picea mariana* was found in fewer sites. More modest but still notable numbers of *Populus balsamifera*, *Abies balsamea*, and *Larix laricina* were encountered on the grid, but these species were found in a more restricted number of sites. *Pinus contorta* Loudon and *Betula papyrifera* Marsh. were found in only 16 and 10 sites respectively and contributed to less than 1% of the total basal area on this landscape.

**Table 2.** Total and relative basal area for species of tree recorded in 194 sites for comparison with beetle assemblage in boreal Alberta, Canada. n= sample size.

Tree species	Basal area (m2)	% basal area	n trees	n sites
*Picea glauca*	82.2298	44.8	1720	133
*Populus tremuloides*	42.6206	23.2	831	91
*Picea mariana*	16.8314	9.2	1655	83
*Populus balsamifera*	36.4818	19.9	541	57
*Abies balsamea*	1.0901	0.6	103	20
*Larix laricina*	3.1918	1.7	86	17
*Pinus contorta*	0.9776	0.5	26	16
*Betula papyrifera*	0.2231	0.1	11	10

A two dimensional NMS solution arrayed the carabid assemblages collected from 193 sites in four quadrants (Fig. 2) with a stress of 15.1. One third of the sites clustered in the upper right quadrant in which values on both NMS axes were positive. The centroids for , *Calathus advena* and *Pterostichus brevicornis* were concentrated in this first quadrant of the ordination diagram, even though these species were captured at a broad range of sites. Centroids for the largest number of abundant species were concentrated in quadrant IV, which included about 25% of the sites. Species in quadrant IV were: *Carabus chamissonis* Fisher von Waldheim, *Pterostichus adstrictus*, *Calathus ingratus*, and with increasingly negative values on axis 2, *Patrobus foveocollis*, *Pterostichus pensylvanicus* LeConte, *Platynus decentis*, *Trechus chalybeus*, *Agonum retractum* and *Agonum sordens* Kirby. Another 25% of the sites were distributed in quadrant II, along with the centroid for *Pterostichus punctatissimus* and the vector for increasing wetness of drainage classes. This vector indicates a concentration of sites with poorly drained soils in the second quadrant. Only 15% of the sites were located in the quadrant III along with the centroids for *Agonum gratiosum* (Mannerheim) and *Platynus mannerheimii* (Dejean). The centroid for *Trechus apicalis* Motschulsky was also placed in quadrant III, although sites with *Trechus apicalis* were also widely distributed in the second and fourth quadrant.

**Figure:**
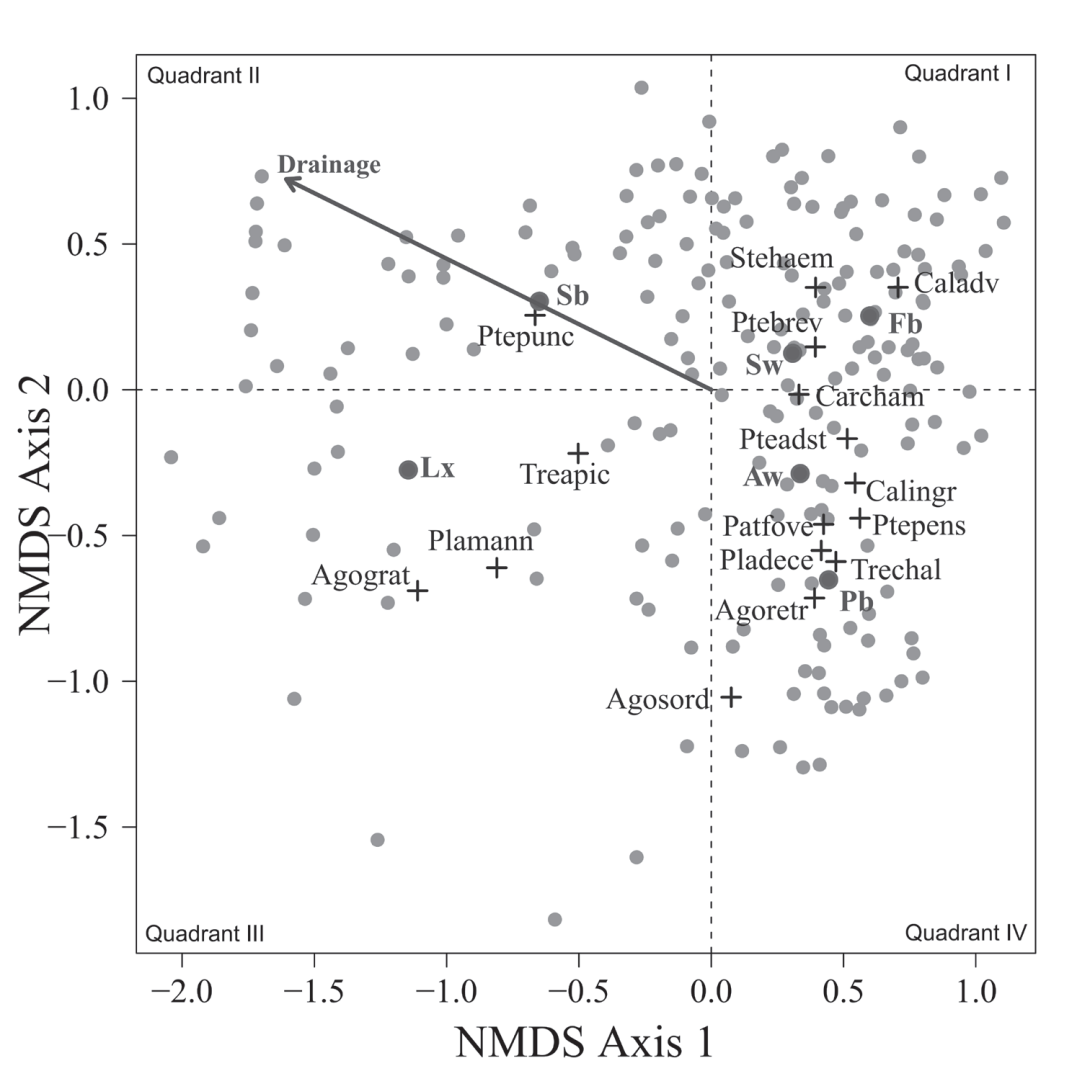
**Figure 2.** NMS ordination of the 193 sites (grey dots) with weighted centroid for beetle (crosses) and tree species (dark dots), stress = 15.1. Vector direction indicates sites with increasingly poor drainage. The abbreviations of the beetle species are as follow: Agograt: *Agonum gratiosum*, Agoretr: *Agonum retractum*, Agosord: *Agonum sordens*, Caladve: *Calathus advena*, Calingr: *Calathus ingratus*, Carcham: *Carabus chamissonis*, Patfove: *Patrobus foveocollis*, Pladece: *Platynus decentis*, Plamann: *Platynus mannerheimmi*, Pteads: *Pterostichus adstrictus*, Ptebrev: *Pterostichus brevicornis*, Ptepens: *Pterostichus pensylvanicus*, Ptepunc: *Pterostichus punctatissimus*, Stehaem: *Stereocerus haematopus*, Treapic: *Trechus* apicalis, Trechal: *Trechus chalybeus*. Abbreviations for the tree species are; Aw: *Populus tremuloides*, Fb: *Abies balsamea*, Lx: *Larix laricina*, Pb: *Populus balsamifera*, Sb: *Picea mariana*, and Sw: *Picea glauca*.

Despite the fact that this ordination was calculated strictly from the beetle data, relative basal area of each tree species is organized in an interpretable pattern when projected into the ordination space of Fig. 2. A detailed depiction of the fit for each tree species is provided in Figs 3 to 8. For example, the highest values of relative basal area for the most abundant tree species, *Picea glauca* (Fig. 3), were clearly concentrated in the first quadrant together with those for *Abies balsamea* (Fig. 4), which was more restricted in relation to beetle sites. Sites with maximum values of relative basal area for *Populus balsamifera* (Fig. 5) were concentrated mainly in the lower part of the fourth quadrant of the ordination, but some intermediate and low values were also encountered in the first quadrant. *Populus tremuloides* was mostly distributed on the right side of the ordination biplot and seems to perform best when sites are defined by beetles that are characteristic of the first quadrant (Fig. 6). The highest values of relative basal area for *Picea mariana* occurredtoward the negative end of the x-axis, especially in the second quadrant (Fig. 7). A few wetter sites placed in the third quadrant were dominated by *Picea glauca* (Fig. 3) and *Larix laricina* (Fig. 8).

Fig. 9 provides a clear depiction of the drainage classes for each site on the ordination diagram. Sites on the right site of the ordination are generally better drained than sites on the left side of the ordination. Among the sites concentrated on the right side of the ordination, drier sites are mostly found below the x axis while mesic sites are found above the x axis.

**Figure:**
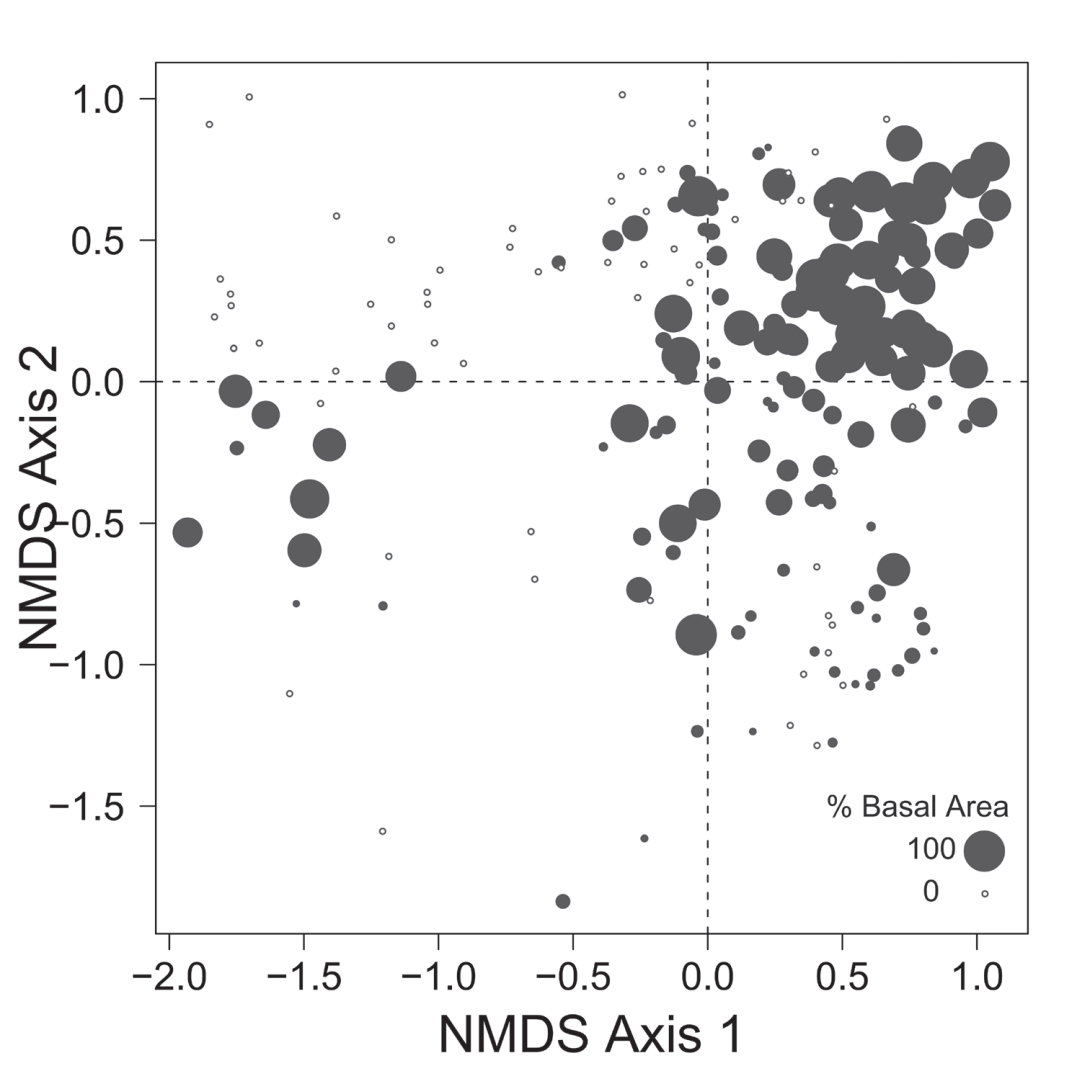
**Figure 3.** Relative basal area of *Picea glauca* for the 193 sites plotted on the beetle ordination of figure 2.

**Figure:**
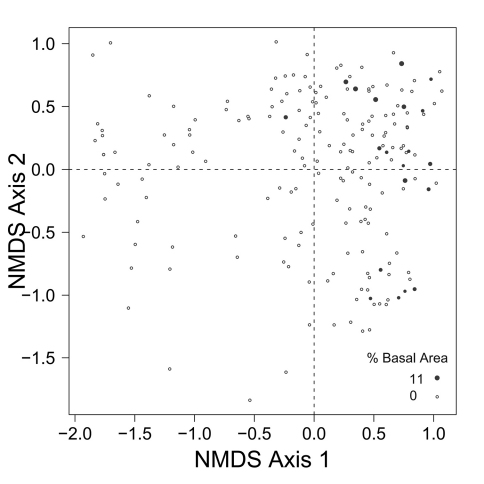
**Figure** **4.** Relative basal area of *Abies balsamea* for the 193 sites plotted on the beetle ordination of figure 2.

**Figure:**
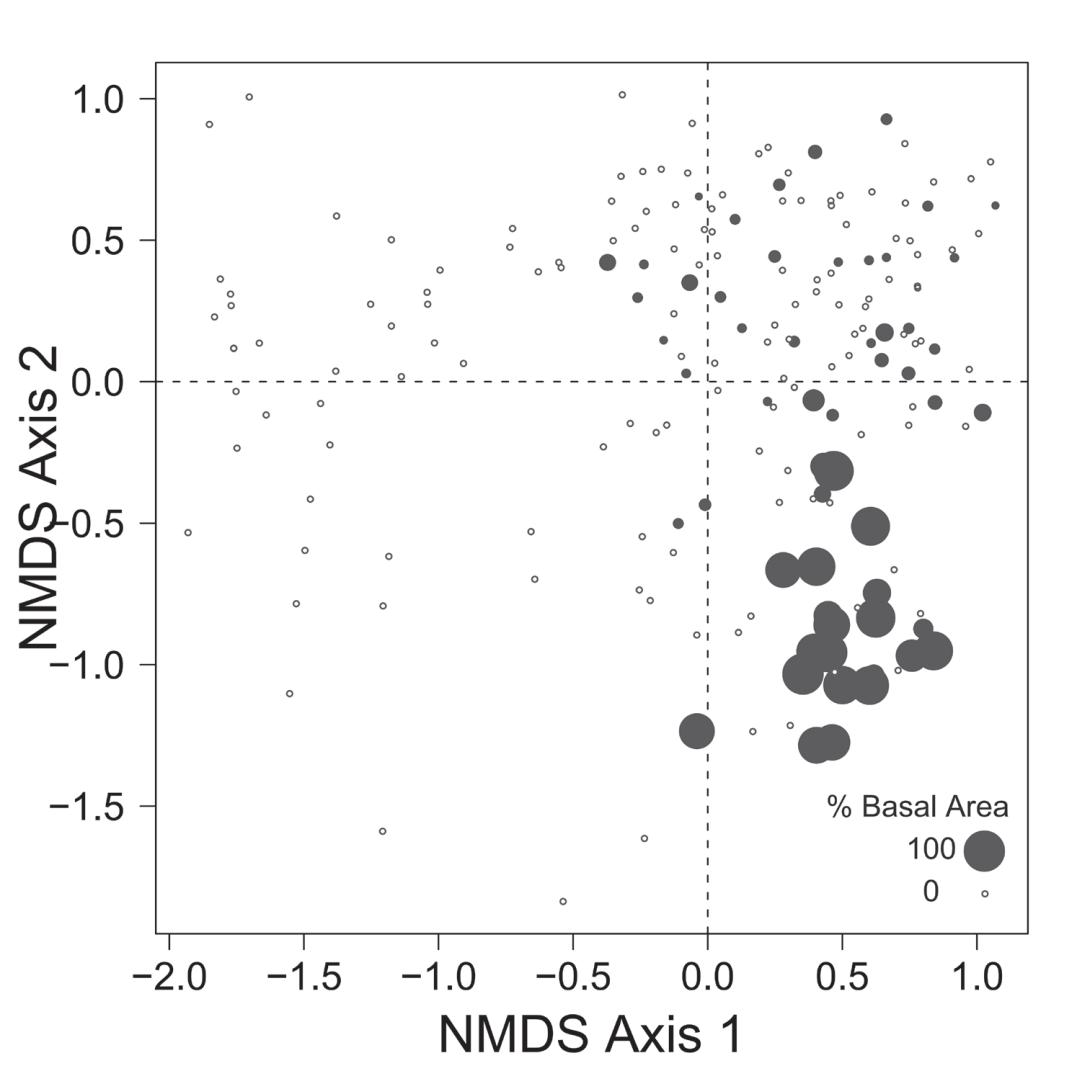
**Figure** **5.** Relative basal area of *Populus balsamifera* for the 193 sites plotted on the beetle ordination of figure 2.

**Figure:**
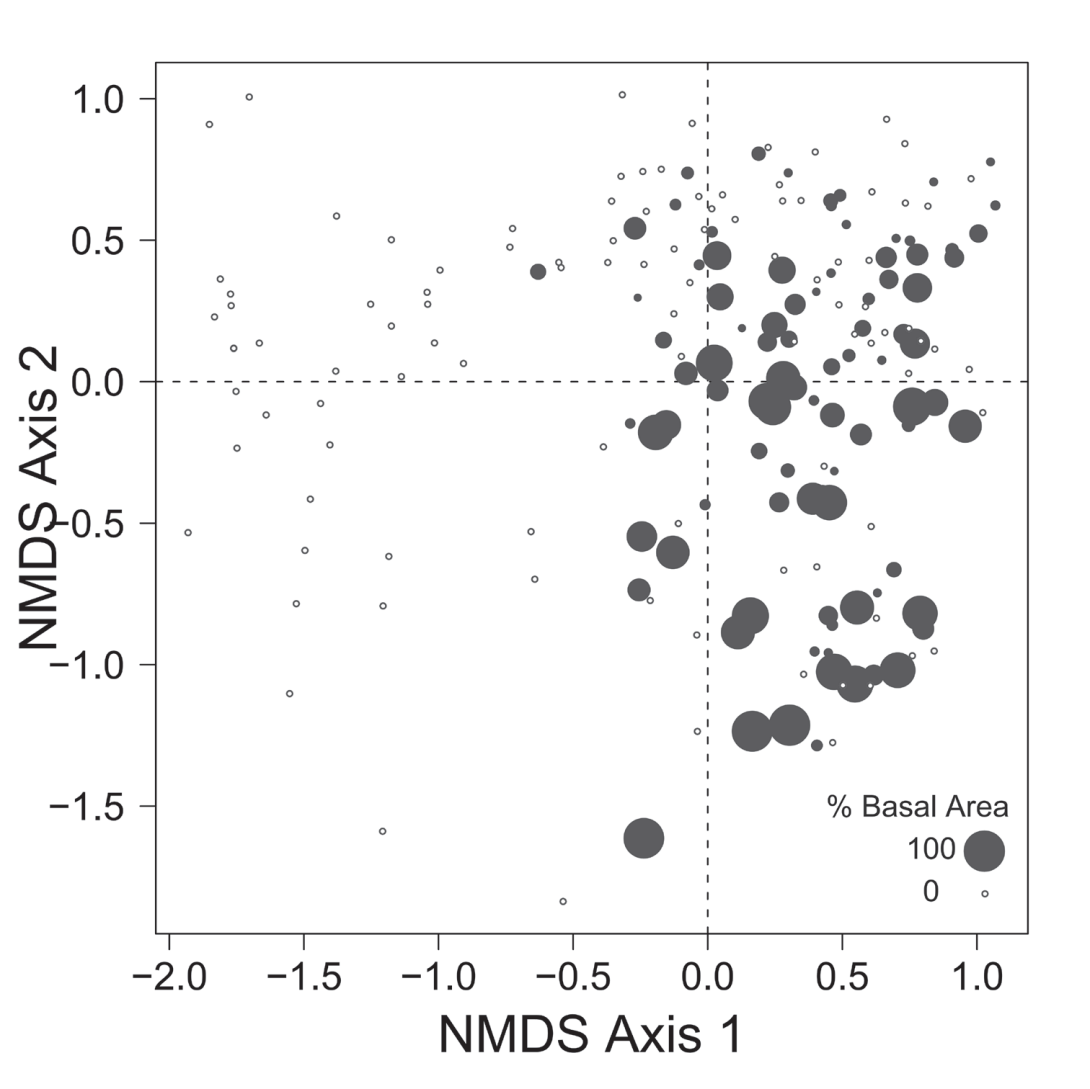
**Figure** **6.** Relative basal area of *Populus tremuloides* for the 193 sites plotted on the beetle ordination of figure 2.

**Figure:**
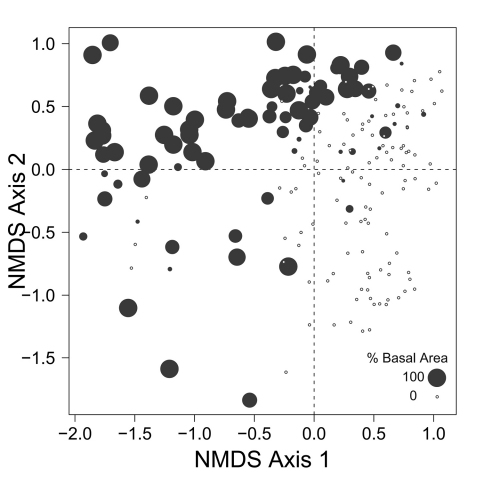
**Figure** **7.** Relative basal area of *Picea mariana* for the 193 sites plotted on the beetle ordination of figure 2.

**Figure:**
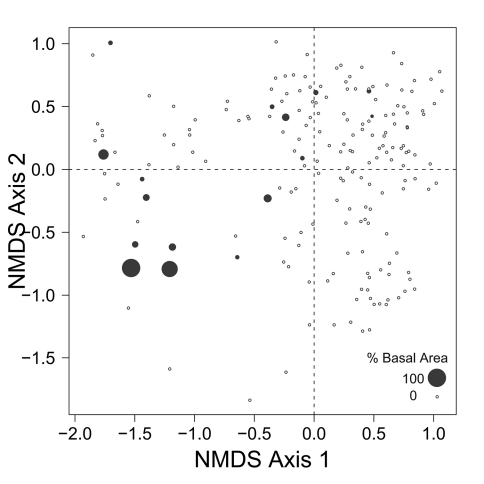
**Figure** **8.** Relative basal area of *Larix laricina* for the 193 sites plotted on the beetle ordination of figure 2.

**Figure:**
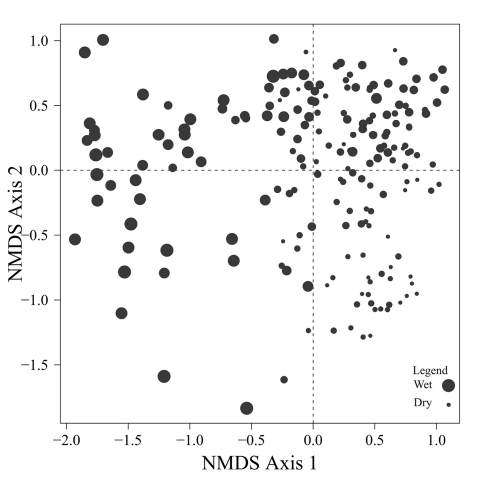
**Figure** **9.** Drainage values for the 193 sites plotted on the beetle ordination of figure 2. High drainage values represent poorly drained sites.

## Discussion

Ground-beetles were collected in all sites, and in numbers large enough to allow robust statistical analysis. This feature alone contributes to the suitability of this beetle family as useful biodiversity indicators for the mixedwood boreal forest mosaic (Rainio and Niemelä 2003). All of the species caught in more than 10 sites (Table 1) are common in and apparently well-adapted to boreal forest conditions; many of them are characteristic of mature and late successional forests (Niemelä et al. 1993; Spence et al. 1996; Jacobs et al. 2008) in Alberta. This result is to be expected, given our sampling design that focused on mature forest that has never been harvested. The ordination data presented here suggest that variation in composition of the forest-associated carabid community is structured by the same environmental factors that affect distribution of the trees, or perhaps is even by the trees *per se*.

Eight of the species trapped only at the disturbed sites (all except *Elaphrus clairvillei*) are characteristic of open habitats (Lindroth 1961-69, Larochelle and Larivière 2003). We captured many additional species also characteristic of open habitats but which constituted less than 0.1% of the total beetle catch (Table 1). Thus, even on boreal landscapes that may be characterized as ‘forested', ample source populations of these species are available to respond to natural forest gaps that become available through disturbance. The EMEND landscape is embedded in a mosaic of harvested and burnt sites that could support such populations, and it seems that individuals of these species even wander into areas that are mainly mature well developed forest.

The main ordination (Fig. 2) represents the landscape according to what portion of the overall beetle assemblage is found in each site. The fact that relative basal area of tree species shows a level of organization in ordination space (Fig. 2) suggests that as the ground-beetle assemblage shifts, the species included exploit different resources, and that availability of these resources vary with presence of particular tree species. It is also interesting to note that tree species group in the ordinations according to ecological similarities documented for these trees. Tree cover on the right side of the ordination, for example, is dominated by a combination of *Picea glauca*, *Populus tremuloides*, *Populus balsamifera*, and *Abies balsamea*. These species are characteristic of uplands in the lower foothills ecoregion of northwestern Alberta, occurring mainly in mesic to well-drained sites either in mixture or pure stands (Beckingham et a. 1996). On the other hand, high relative basal areas of *Picea mariana* and *Larix laricina*, species that typically colonize wet lowlands, are found mostly on the left side of the ordination. The drainage vector projected on the ordination diagram (Fig. 2) confirms this major ecological difference between species on the right and left side of the ordination. All eleven carabid species representing over one percent of the collection (Table 1) have their centroids located on the right side of the ordination, except *Pterostichus punctatissimus* (Fig. 2). This underscores the general inhospitality of very poorly drained sites for ground-beetles, an interesting generalization from this study. Organization of the beetle community seems to follow general ecological requirements of tree species, and thus, soil drainage is one of the factor that drives the distribution of both tree and beetle species.

The distribution of carabids on the landscape may be explained in even more detail by isolating the connections to particular tree species in relation to what is known about habitat use of these carabids. For example, the beetle species arrayed around the centroid for *Populus balsamifera* (*Agonum retractum*, *Trechus chalybeus*, *Platynus decentis*, *Patrobus foveocollis*, and *Pterostichus pensylvanicus*; see Fig. 2) typically prefer moderately moist ground (Larochelle and Larivière 2003). In contrast, these authors note that beetle species located near the centroid for *Populus tremuloides* (*Calathus ingratus* and *Pterostichus adstrictus*) in Fig. 2 prefer drier ground than the aforementioned species. Despite similar ecology, *Populus balsamifera* occupies wetter sites than *Populus tremuloides* (Burns and Honkala 1990) and this is reflected in the distribution of their relative basal areas according to beetle species (Fig. 2). Changes in the beetle assemblage along this gradient are strong enough to suggest differential influences on the structure of ground-beetle community by these two ecologically similar tree species.

*Calathus ingratus* and *Pterostichus adstrictus* are considered as habitat generalists in non-riparian areas of the boreal zone (Niemelä et al. 1993, Pearce and Venier 2006) and are found here along most of the deciduous-coniferous gradient on the right side of the ordination. The highest abundances of *Carabus chamissonis* are also found to occur on drier grounds. Being characteristic of mixed and coniferous forest (Larochelle and Larivière 2003), most *Carabus chamissonis* were trapped at transitions between the deciduous and coniferous components of this gradient (Fig. 2), a new finding in this study.

Sites located in the first quadrant are dominated by high values of relative basal area for *Picea glauca* and *Abies balsamea*, and are associated with high abundances of *Stereocerus haematopus*, *Calathus advena*, and *Pterostichus brevicornis* (Fig. 2). All three of these species are regularly associated with coniferous forest (Spence et al. 1996, Gandhi et al. 2001, Work et al. 2004). *Calathus advena* is most frequently trapped in the forest, but both *Stereocerus haematopus* and *Pterostichus brevicornis* may occur in more open spruce bogs or heaths with ericaceous vegetation characteristic of higher altitude and latitude (Lindroth 1966). This explains why in our ordination, the centroids for these two later species are located closer to the y-axis, where the presence of *Picea glauca* and *Picea mariana* overlap (Fig. 3 and 7). Despite the fact that *Stereocerus haematopus* and *Pterostichus brevicornis* may occur in habitat where black spruce grow, they are normally encountered on drier ground than is *Pterostichus punctatissimus*.

Highest abundances of *Pterostichus punctatissimus* are concentrated in the second quadrant together with the highest relative basal area values for *Picea mariana* (Fig. 2). *Pterostichus punctatissimus*, *Agonum gratiosum*, and *Platynus mannerheimii* are all recognized to occur in coniferous forest (Lindroth 1966, Larochelle and Larivière 2003), especially that dominated by *Picea mariana* (Holliday 1991, Niemelä et al. 1992, Pearce et al. 2003, Paquin 2008). However, both *Platynus mannerheimii* and *Agonum gratiosum* are especially common in wet productive sphagnum bogs, swamps and lowland forested sites dominated by *Picea* and *Larix* (Larochelle and Larivière 2003). In the lower foothills of northwestern Alberta, *Larix laricina* tends to occur in lowlands together with *Picea mariana*; however presence of tamarack indicates productive sites where nutrients are more available (Beckingham et al. 1996). Composition of the beetle assemblage seems to reflect this ecological difference as high abundances of *Platynus mannerheimii* and *Agonum gratiosum* are located in the third quadrant overlapping strongly with the highest relative basal area values of *Larix laricina*.

Lindroth (1963) characterizes *Trechus apicalis* as a eurytopic species with affinities for *Sphagnum*. Although, catches of this beetle are widely distributed on the ordination diagram, most catches occurred to the left side of the ordination (Fig. 2) together with *Picea mariana*, the tree species that dominates *Sphagnum* bogs in the area (Beckingham et al. 1996).

*Agonum sordens* is characterized as hygrophilous (Lindroth 1966), often occurring close to water especially eutrophic marshes. It was placed in the lower part of the fourth quadrant of the ordination together with beetle species characteristic of moist soils and significant cover of *Populus balsamifera*. However, sites with *Agonum sordens* also occurred in the third quadrant together with populations of *Agonum gratiosum* and *Platynus mannerheimii* where beetle assemblage is more characteristic of wet areas. These sites also included beetles more characteristic of upland forest such as *Platynus decentis*, *Trechus chalybeus* and *Pterostichus brevicornis*. These species are characteristic of ‘old growth' forest in Alberta (Spence et al. 1996, Niemelä 1997), and the fact that tree cover at these sites consists of a mix of *Picea mariana*, *Populus tremuloides* and *Picea glauca* likely reflects that a fine-grained mix of upland mesic and lowland wet sites is common to boreal stands in this region. Such subtle local variation certainly contributes to the diversity of epigaeic invertebrates in naturally occurring boreal stands. Maintaining such subtle variation is not an obvious feature of silvicultural practices used to regenerate boreal stands. This also speaks to the need to account for variation in physiography within stands in designing systems to conserve biodiversity.

Despite the fact that ecological linkage between the beetle assemblage and the canopy trees is well depicted using species centroids projected on the beetle ordination (Fig. 2), much ecological information remains hidden. Examination of Figs 3 to 6 reveals that *Picea glauca*, *Populus tremuloides*, *Populus balsamifera*, and *Abies balsamea*, each occupies a wide range of sites on the right side of the ordination corresponding to upland forest. In the boreal mixedwood forest, the beetle community seems not to respond to the habitat as a mixture of pure coniferous and deciduous stands, but rather, as stands supporting a gradual mixture of conifer and deciduous tree species. In the ordination space, sites for coniferous, mixed and deciduous forest are not tightly grouped according to these categories but are evenly dispersed along this gradient. This provides evidence that the ground-beetle community dynamics on this sort of landscape behaves more like one loosely integrated Gleasonian community instead of tight Clementsian species groups showing similar responses to resource distribution.

There is additional evidence that projecting centroids onto the ordination diagram fails to capture some significant ecological patterns. A few sites with high values of relative basal area for *Picea glauca* also group together at the lowest values of the x axis of the NMS ordination (Fig. 3). This is intriguing as these sites seem to host a very different beetle assemblage than most sites with high relative basal area of white spruce. The beetle catches were dominated by *Pterostichus punctatissimus* and *Agonum gratiosum*, but did not include either *Stereocerus haematopus* or *Calathus advena*, species that were characteristic of all other sites with high relative basal area of *Picea glauca* (Fig. 2). In these exceptional sites, *Picea glauca* (Fig. 3) grows with *Larix laricina* (Fig. 8) but the presence of *Picea mariana* that generally supports a beetle community characteristic of wet sites is less important (Fig. 7). In these circumstances, the carabid community is more typical of wet productive sites. Figure 9 combined with the results shown in Figure 3 confirm that white spruce occurring in the most poorly drained sites support a different assemblage than white spruce occurring on moderately to well-drained sites. Following the widely used approach of projecting *Picea glauca* distribution onto the ordination with a vector or, simply projecting the centroid for this species on the ordination diagram (Fig. 2), does not reveal this pattern. We suggest that this is evidence that non-linear gradients can affect arthropod community structure. Before such gradients can be studied and understood, they must be first revealed, something accomplished here by ordination in relation to basal area.

Patterns of association between beetle community structure and uncommon tree species having a restricted distribution on the landscape are of special interest in a conservation context. For example, *Abies balsamea* is at the northwestern edge of its continental range at EMEND and stands are scattered and restricted to narrow habitats (Halliday and Brown 1943, Bakuzis and Hansen 1965), often located in areas “skipped” repeatedly by historical fires (Sirois 1997). The carabid assemblage associated with this tree is a subset of that characteristic of *Picea glauca* stands (Fig. 3 and 4). Both of these tree species are shade tolerant and typical of late-successional forest (Burns and Honkala 1990). Accordingly, two species of beetle that occur together with *Abies balsamea* in the first quadrant (*Calathus advena* and *Pterostichus brevicornis*, Fig. 2) are characteristic of old-growth forest (Niemelä et al. 1993, Jacobs et al. 2008). Furthermore, *Pterostichus brevicornis* appears to be restricted to moist and cool forest areas, such as the interior of sites skipped by fire (Spence et al. 1996). In our ordination, *Abies balsamea* also occurs more marginally in the lower part of the fourth quadrant where the beetle assemblage is characteristic of sites dominated by *Populus balsamifera* (Fig. 4). This may be attributed to the fact that *Populus balsamifera* grows on moist sites (Burns and Honkala 1990), a feature rendering these sites more likely to escape fire and develop the specific edaphic conditions required by *Abies balsamea*. It is interesting to note that these sites also support populations of *Trechus chalybeus* and *Platynus decentis*, species also recognized as typical of old forest (Spence et al. 1996). This interesting trend would also not have been detected simply by plotting centroids or projecting vectors.

A similar pattern of association appears between *Larix laricina* and *Platynus mannerheimii* (Fig. 2), both of which are uncommon species of the EMEND landscape (Table 1 and 2). *Platynus mannerheimii* is generally recognized in both North America and Scandinavia as being an uncommon element of the boreal beetle community, having narrow microhabitat requirements and being locally restricted to mires, old wet forests and fire skips (Niemelä 1997, Haila et al. 1994, Niemelä et al. 1992, Paquin 2008, Gandhi et al. 2001). The strong association between uncommon tree and beetle species suggests that more careful consideration of the distribution and abundance of rare tree species on a landscape would be useful as a coarse-filter for conservation efforts to manage some elements of boreal biodiversity. Presence of these tree species should not be dismissed as ‘noise' in managing a landscape mosaic of commercially important species valued for fiber.

In general, associations between carabid and tree species, as described previously, match species of the same rank order of abundance on the EMEND landscape (Table 1 and 2). For example, the beetle species collected in the highest number of sites (*Stereocerus haematopus* and *Calathus advena*) were associated with *Picea glauca*, the tree species similarly recorded from the highest number of sites. This is also true for the association between the carabids *Pterostichus adstrictus* and *Calathus ingratus* and the tree species, *P .tremuloides* (second highest number of sites). *Pterostichus punctatissimus*, the sixth most common carabid, was associated with *Picea mariana* (noted at the third highest number of sites). Likewise *Agonum retractum*, *Trechus chalybeus*, and *Patrobus foveocollis* were associated with *Populus balsamifera* (fourth highest number of sites), and *Pterostichus brevicornis* and *Carabus chamissonis* were predominately collected at sites with *Abies balsamea* (fifth highest number of sites). As outlined above, *Agonum retractum* and *Platynus mannerheimii* were associated with *Larix laricina* (recorded at the sixth highest number of sites). We suggest that each tree species indicates its own set of edaphic and perhaps even broader environmental conditions. If so, beetle species requiring conditions related to the most frequent tree encountered on a landscape will also be the most commonly encountered in systematic sampling efforts to the extent that beetle population sizes follow that of tree species. If a habitat is less frequent on a landscape, the beetle requiring this habitat should also be less frequently collected, and this is what we observed. Thus, including all tree species in stand-level forest inventories can have real practical value in developing regional conservation strategies.

## Conclusion

Despite the indirect nature of potential links between distributions of tree and ground-beetle species, the ecological features associated with their distributions appear to be similar, allowing us to discern surprisingly clear patterns of association. Variation in carabid assemblages over this section of the boreal forest reflects the specific presence of all tree species present on this landscape. It is unknown at present the extent to which these associations simply reflect a response to common features or if, perhaps, the trees themselves contribute to conditions (e.g., through quality of litter) that promote success of particular invertebrate species. Furthermore, although associations between ground-beetle and tree species are strong and interpretable, the potential implication for predicting distribution of other invertebrate taxa remains to be investigated.

Nevertheless, these observations are of interest for regional conservation purposes. Because the boreal forest covers vast areas, it is impossible in cost-effective practice to assess biodiversity reliably, and thus surrogates are needed (Spence et al. 2008). Our results support developing an approach that uses details of forest inventory as a possible surrogate for arthropod biodiversity in conservation planning. Extensive Canadian forest surveys already include evaluation of canopy cover (Leckie and Gillis 1995), and this can be directly related to basal area (Spurr 1960) as used in this study. Our study emphasizes that accuracy of forest surveys and inventories is crucial to their use in conservation planning because uncommon carabid species are clearly associated with uncommon tree species. It will be important to include records of these uncommon tree species in all inventories and to ensure that methods employed in regeneration of forests managed extensively permit such trees to establish themselves in a rather natural manner, even when they are commercially unimportant.
